# Ileo-Colonic Fistula Due to Diffuse Large B Cell Lymphoma: Unusual Presentation of a Rare Disease

**DOI:** 10.7759/cureus.12956

**Published:** 2021-01-28

**Authors:** Godson Senyondo, Saif Bella, Atif Saleem, Sarah A Mehdi, Jagmohan Sidhu

**Affiliations:** 1 Internal Medicine, Wilson Memorial Hospital, Johnson City, USA; 2 Gastroenterology and Hepatology, Wilson Memorial Hospital, Johnson City, USA; 3 College of Medicine, Upstate University Hospital, Syracuse, USA; 4 Pathology and Laboratory Medicine, Wilson Memorial Hospital, Johnson City, USA

**Keywords:** primary gastro intestinal lymphoma, malignant ileo-colonic fistula.

## Abstract

Malignant ileocolonic fistulas have seldom been documented as complications of a primary gastrointestinal lymphoma (PGIL) such as aggressive diffuse large B cell lymphoma (DLBCL). These fistulas are frequently misdiagnosed due to the nonspecific clinical presentation. Currently, there is no standardized treatment approach, although a couple have been suggested with varying outcomes. We describe a case of DLBCL complicated with a malignant ileocolonic fistula in a 55-year-old male with a favorable outcome after surgery and chemotherapy.

## Introduction

This article was previously presented as a meeting abstract at the Annual American College of Gastroenterology (ACG) 2020 virtual Scientific Meeting on October 23, 2020.

Primary gastrointestinal lymphoma (PGIL) is a rare malignancy that proves to be a diagnostic challenge due to the nonspecific clinical presentation and may easily be misdiagnosed as Crohn’s disease or intestinal tuberculosis [1,2]. Diffuse large B cell lymphoma (DLBCL) is the most common histologic subtype of non-Hodgkin’s lymphoma (NHL) with a male predominance of 55% and a median age of 50-60 years [3]. Although mostly a nodal disease, 40% of cases of DLBCL involve extranodal sites such as the gastrointestinal tract [4,5]. Tumor intestinal fistulas are rare but serious complications of advanced primary intestinal lymphoma like DLBCL, with a paucity of reported cases [6,7]. We present a case of an ileo-colonic fistula due to primary DLBCL of the ileum in a 55-year-old male, followed by a case discussion and literature review.

## Case presentation

**Figure 1:**
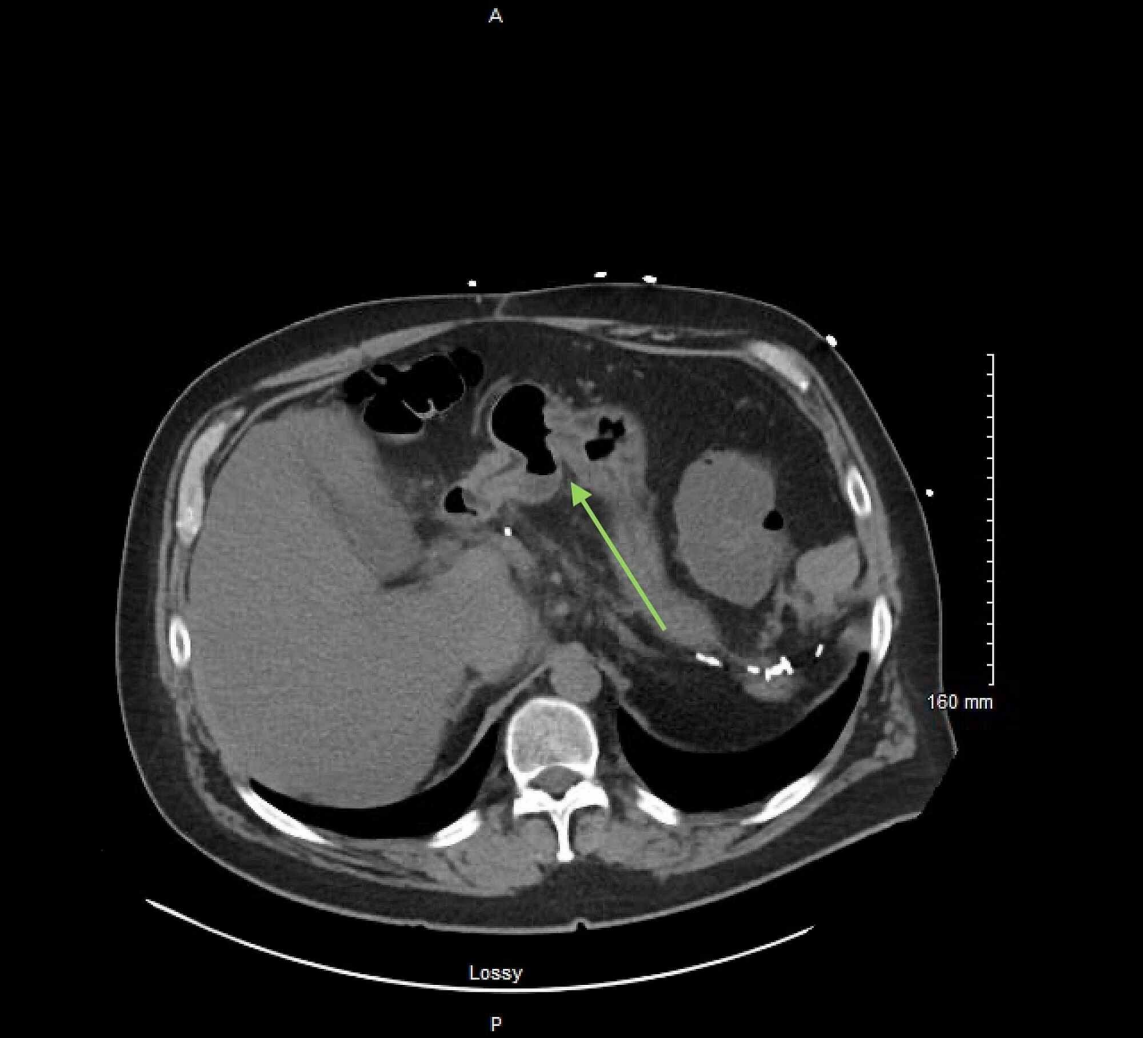
Unenhanced cross section CT scan of the abdomen showing an ileocolonic fistula with aneurysmal dilation of the small bowel (green arrow).

A 55-year-old male presented to the emergency room complaining of three months of watery, non-bloody, non-melanotic diarrhea with generalized abdominal pain for three weeks and nausea with constant gurgling “washing machine” sounds. He denied any vomiting, fever, chills, anorexia, or weight loss. His medical history was significant for hypothyroidism and Hodgkin's lymphoma 25 years ago, which required a staging laparotomy with splenectomy and was treated with mantle field radiation therapy for a cure. The physical exam, including abdominal examination, was unremarkable. Of note, his labs revealed elevated C-reactive protein (CRP) 15.3mg/dL and leukocytosis of 14.3K/uL, but were otherwise within normal limits. Non-contrast CT scan of the abdomen and pelvis showed a focal aneurysmal dilation of a loop of small bowel in the mid-lower abdomen with several enlarged mesenteric lymph nodes, and a fluid-filled sigmoid colon (Figure 1).

**Figure 2:**
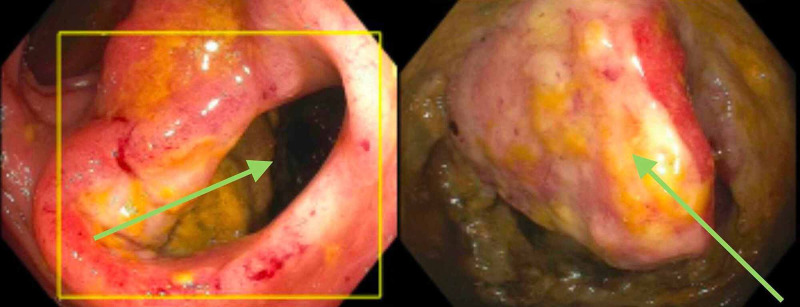
Endoscopic picture showing the fistula at 20cm from the anal verge (left) and a small bowel fistulizing mass (right).

These findings were concerning for a small bowel tumor, with a suspected entero-colonic fistula. Subsequently, a colonoscopy was performed, confirming a large sigmoid colon fistula at 30 cm from the anal verge that communicated with the midgut lumen through a medium-sized ulcerated mass (Figure 2, Video 1).

**Video 1:** The Ileo-colonic fistula as seen on colonoscopy.

**Figure 3:**
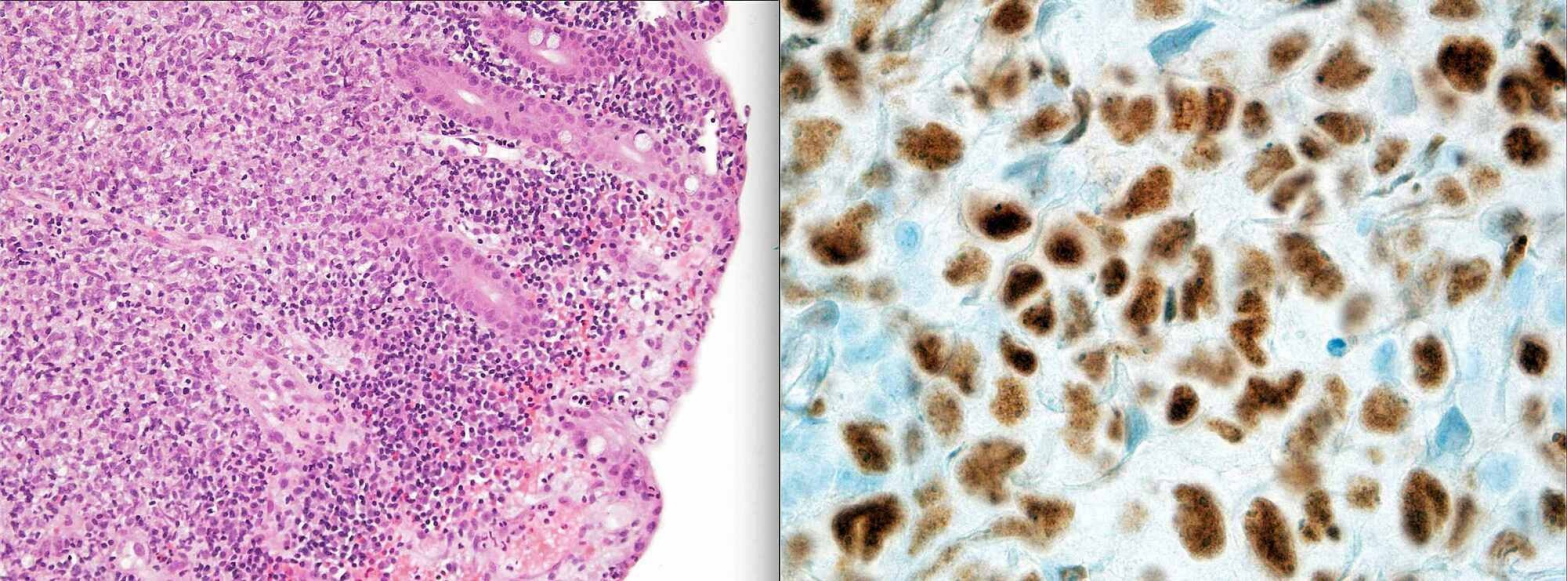
H&E (left) and BCL6 (right) stained histology slides showing malignant high grade DLBCL cells (MYC and IGH/BCL2 negative but BCL6 positive) infiltrating the small bowel. BCL6: B-cell lymphoma 6; H&E: hematoxylin and eosin; DLBCL: diffuse large B-cell lymphoma

The patient underwent an exploratory laparotomy with en bloc excision of the mass and fistula. Both colonoscopy and surgical histopathology revealed a high grade aggressive primary DLBCL, germinal center-like (Figure 3).

Bone marrow biopsy was negative for malignancy, but whole-body PET/CT scan revealed hypermetabolic left para-aortic and right mid abdomen lymph nodes concerning for a persistent neoplastic process prompting the use of standard adjuvant chemotherapy for DLBCL consisting of rituximab, vincristine, etoposide, cyclophosphamide, and prednisolone (R-COEP). Doxorubicin was avoided in view of the patient's ventricular ejection fraction (EF) of 40-45%. Repeat PET/CT scan after the sixth chemotherapy cycle showed decreased size of the lymph nodes with resolution of their hypermetabolic state and no acute bowel pathology or metastatic disease. The patient was declared in remission with a satisfying clinical outcome. 

## Discussion

Malignant ileo-colonic fistula is occasionally associated with adenocarcinoma of the cervix, ovaries and colon [7,8]. In 1862, the first case of malignant gastrointestinal fistula secondary to a cecal adenocarcinoma was reported, but it was not until 1946 that a fistula due to primary gastrointestinal (GI) lymphoma was described [9,10].

Small bowel lymphoma is reported as the cause of less than one percent of all gastrointestinal malignancies, and in the absence of prior chemotherapy, surgical resection, or recent radiotherapy, the development of fistula is extraordinarily rare [7]. Nine primary lymphoma cases causing entero-colic fistula have been reported in literature; six are of B cell origin and three of T cell origin [6,7,11].

The formation of a malignant fistula is a chronic process that occurs over an extended interval [12]. The lymphoma is initially confined to the submucosa but extends into and invades through the serosa and nearby structures through coagulative necrosis, ulceration, and liquefaction of tissue [12]. It penetrates the mesentery and drains into the adjacent tissue, resulting in abscesses in the peritoneal cavity, or fistula formation in the adjacent intestinal wall [1,12,13].

The nonspecific symptoms of GI fistula delay diagnosis, with the most common presenting symptoms being abdominal pain (84%), weight loss (81%), and diarrhea (39%). Twenty-three percent of patients present with small bowel perforation [14]. The aneurysmal dilation seen in our patient has been hypothesized to be due to tumor invasion of nerve plexus causing reduced muscular tension [10]. Intractable diarrhea was seen with our patient. In literature, it has been hypothesized to be either secondary to small intestine bacterial overgrowth from colonic microbiota; or a phenomenon similar to short bowel syndrome where the fistula allows intestinal contents to empty directly into the colon prematurely [15]. Unlike most of the reported cases, our patient did not report any weight loss. This may be due to the aggressive course of DLBCL, allowing for prompt diagnosis.

Although PET/CT is instrumental in detecting PGIL, defining the degree of metastasis, stage, and subsequent treatment response, our patient required a colonoscopy to confirm the ileocolonic fistula suspected on the abdominal CT imaging. There is currently no unified consensus on the optimal treatment strategy for intestinal lymphoma fistulas [16]. Zhuang et al. described a case of DLBCL with an ileo-sigmoid fistula treated with eight cycles of standard chemotherapy and no surgical intervention, with complete resolution of fistula on repeat CT imaging [6]. Milburn et al. piloted a novel approach that employed endoscopic duodenal stenting, allowing for the early introduction of chemotherapy that successfully resolved the fistula [17]. The method we implemented for our patient is the commonly reported treatment approach. It involves surgical resection of the tumor, fistula and adjacent lymph nodes for accurate tissue diagnosis and local intraoperative staging of the malignancy. This is then followed by standard adjuvant chemotherapy consisting of R-CHOP [16.17]. Our patient was treated with the alternative chemotherapy of R-CEOP, avoiding doxorubicin due to a relatively low EF of 40-45%. He had an excellent outcome with complete resolution of symptoms and was declared disease free after six cycles of chemotherapy.

## Conclusions

Ileo-colonic fistula due to intestinal lymphoma is a rare occurrence. Due to nonspecific clinical presentation, a high index of suspicion is required to establish the diagnosis of ileo-colonic fistula. Besides, we recognize colonoscopy's role in confirming the ileo-colonic fistula diagnosis if the diagnosis is not clear on CT imaging. Currently, there is no consensus on a standardized treatment approach, but we recognize the approach of en bloc surgical resection of the tumor followed by standard adjuvant chemotherapy as the best treatment approach to ileo-colonic fistulas. However, we admit that more data is required to demonstrate the most effective approach to PGIL with fistula. We hope this case serves to assist in early detection and guiding treatment to pursue the optimal clinical outcomes for patients with PGIL complicated by fistula.
